# Nitrogen Substrate Utilization in Three Rhizosphere Bacterial Strains Investigated Using Proteomics

**DOI:** 10.3389/fmicb.2020.00784

**Published:** 2020-04-28

**Authors:** Richard P. Jacoby, Antonella Succurro, Stanislav Kopriva

**Affiliations:** Institute for Plant Sciences and Cluster of Excellence on Plant Sciences (CEPLAS), University of Cologne, Cologne, Germany

**Keywords:** nitrogen, bacteria, metabolism, amino acids, proteomics, rhizosphere microbiome, flux balance analysis, phenotype microarray

## Abstract

Nitrogen metabolism in the rhizosphere microbiome plays an important role in mediating plant nutrition, particularly under low inputs of mineral fertilizers. However, there is relatively little mechanistic information about which genes and metabolic pathways are induced by rhizosphere bacterial strains to utilize diverse nitrogen substrates. Here we investigate nitrogen substrate utilization in three taxonomically diverse bacterial strains previously isolated from Arabidopsis roots. The three strains represent taxa that are consistently detected as core members of the plant microbiome: Pseudomonas, Streptomyces, and Rhizobium. We use phenotype microarrays to determine the nitrogen substrate preferences of these strains, and compare the experimental results vs. computational simulations of genome-scale metabolic network models obtained with EnsembleFBA. Results show that all three strains exhibit generalistic nitrogen substrate preferences, with substrate utilization being well predicted by EnsembleFBA. Using label-free quantitative proteomics, we document hundreds of proteins in each strain that exhibit differential abundance values following cultivation on five different nitrogen sources: ammonium, glutamate, lysine, serine, and urea. The proteomic response to these nitrogen sources was strongly strain-dependent, with lysine nutrition eliciting widespread protein-level changes in *Pseudomonas* sp. *Root9*, whereas *Rhizobium* sp. *Root491* showed relatively stable proteome composition across different nitrogen sources. Our results give new protein-level information about the specific transporters and enzymes induced by diverse rhizosphere bacterial strains to utilize organic nitrogen substrates.

## Introduction

Improved nitrogen management in agricultural systems is crucial for environmental sustainability. Large-scale application of mineral nitrogen fertilizers has extensive off-target effects, such as greenhouse gas production and waterway eutrophication (Zhang et al., 2015). One potential pathway to boost agricultural sustainability involves substituting mineral fertilizers with organic nutrients derived from recycling various waste streams. For low-input agricultural systems to provide sufficient bioavailable nitrogen to meet the demands of plant growth, future crop management practices will need to better incorporate microbial pathways of nitrogen mobilization (Hirsch and Mauchline, 2015). One specific suggestion involves engineering the rhizosphere microbiome to promote the mineralization of organic nitrogen, coupled with engineering of plant root metabolism to release rhizodeposits that recruit beneficial microbial strains (Bender et al., 2016). However, the ability to manipulate plant-microbe cooperation is limited by an incomplete knowledge of the specific microbial traits involved in root colonization and nutrient mobilization (Trivedi et al., 2017).

Nitrogen flows in the rhizosphere are complex, with plants and microbes potentially cooperating but sometimes competing for uptake of diverse nitrogen molecules (Bloom, 2015). Legume-Rhizobia symbioses provide an example of cooperation, whereby the majority of the plant’s nitrogen nutrition is derived from bacterial fixation of atmospheric N_2_ (Peoples et al., 2009). Outside of legumes, it is generally accepted that plants obtain the majority of their nitrogen nutrition from inorganic forms such as nitrate and ammonium, whereas microbes are more adept at acquiring more recalcitrant organic nitrogen forms such as proteins and amino acids (Kuzyakov and Xu, 2013). Therefore, cooperative nutrient transfers can occur when microbes take up soil-bound organic nitrogen, which is subsequently transferred to plants in a mineralized form following microbial lysis or protozoic predation (Harrison et al., 2007). Conversely, competitive flows can occur when microbes immobilize inorganic nitrogen, or when plants take up organic nitrogen (Jones et al., 2013). Adding further complexity, plant root exudates contain large amounts of organic nitrogen molecules which can serve as carbon and nitrogen substrates for bacterial growth. The rate of amino acid release from plant roots increases under exposure to specific bacterial metabolites (Phillips et al., 2004), but organic nitrogen molecules released via root exudation can also be efficiently re-acquired by the root system (Warren, 2015).

Investigations of how bacteria utilize diverse nitrogen substrates have been documented since the beginning of modern microbiology (Koser and Rettger, 1919). Ammonium is the preferred nitrogen source for most bacteria, and experimental designs usually include ammonium as a control treatment, to compare against alternative nitrogen sources or starvation treatments (Merrick and Edwards, 1995). Over decades, such studies have provided detailed insight into fundamental physiological mechanisms such as the molecular pathways of bacterial nitrogen assimilation, the perception of nitrogen status, and the response to nitrogen starvation in *E. coli* (van Heeswijk et al., 2013). However, Gram positive bacteria possess different mechanisms for regulating nitrogen metabolism (Fisher, 1999; Amon et al., 2010), and soil bacteria exhibit considerable extensive diversity regarding their nitrogen substrate preferences and also the metabolic pathways used to metabolize organic nitrogen sources (Geisseler et al., 2010; Moe, 2013). Therefore, novel insights into metabolic mechanisms of nitrogen metabolism may be observed by studying nitrogen substrate utilization in taxonomically diverse bacterial strains isolated from the rhizosphere.

The rhizosphere microbiome has attracted increasing research attention over the past 20 years. From the results of 16S pyrosequencing studies, it has become increasingly apparent that the rhizosphere hosts a taxonomically diverse bacterial microbiota, which plays an important role in determining plant growth and health (Muller et al., 2016). Recently, multiple research groups have established large collections of bacterial strains isolated from field-grown plants, which can be used to dissect the functional traits carried out by individual strains, or reassembled into synthetic communities that recapitulate microbiome function (Bai et al., 2015; Levy et al., 2018b; Gomez-Godinez et al., 2019). There is now an opportunity to study these plant-associated microbial strains using high-throughput omics techniques, to acquire new insights into the specific molecular mechanisms that confer a selective advantage in the plant-associated niche (Levy et al., 2018a).

Alongside experimental approaches, computational modeling is becoming a widespread approach to investigate microbial metabolism (Zomorrodi and Segre, 2016). One particularly useful method is the construction of genome-scale metabolic network models, which translate the information encoded in the bacterial genome into a computational formalism that can be analyzed with mathematical methods (Henry et al., 2010). However, curated genome-scale metabolic models are only available for a relatively small set of extensively studied bacterial strains, and generally it is difficult to analyze newly sequenced bacterial strains using computational modeling. This limitation exists because reconstructing a curated genome-scale metabolic network model is a painstaking process that requires extensive manual curation as well as the acquisition of extensive experimental data, particularly regarding biomass composition. Although progress is being made toward automated reconstruction of genome-scale metabolic network models, many challenges still have to be addressed (Arkin et al., 2018). Recently, a method named EnsembleFBA has been proposed as a potential approach to approximate genome-scale metabolic networks for diverse bacterial strains. Instead of relying on the availability of a single manually curated genome-scale model, EnsembleFBA uses the information derived from multiple metabolic networks, which are reconstructed from the same initial draft network and refined through the process of positive and negative gapfilling on randomized sets of growth and non-growth conditions. As a proof of concept, it was shown that the EnsembleFBA method achieved greater precision in predicting essential genes than an individual, highly curated model (Biggs and Papin, 2017).

Here we investigate nitrogen metabolism in three taxonomically diverse bacterial strains previously isolated from Arabidopsis roots. We apply a combination of methods, including quantitative proteomics, growth assays, phenotype microarray, and EnsembleFBA. With the proteomic data, we were particularly interested in determining the specific proteins that are enriched according to different nitrogen sources, to decipher the metabolic strategies used for nitrogen acquisition across different rhizosphere bacterial strains. In parallel, we applied the EnsembleFBA method to reconstruct and analyze sets of genome-scale metabolic network models for each strain, using the phenotype microarray data for training and testing the model predictions of nitrogen substrate utilization.

## Materials and Methods

### Bacterial Strains

Bacterial strains used in this study were *Pseudomonas* sp. *Root9* (NCBI Taxonomy ID: 1736604), *Streptomyces* sp. *Root66D1* (NCBI Taxonomy ID: 1736582) and *Rhizobium* sp. *Root491* (NCBI Taxonomy ID: 1736548), all isolated from field-grown Arabidopsis roots (Bai et al., 2015) and provided by Paul Schulze-Lefert, MPIPZ Cologne.

### Bacterial Pre-cultivation and Harvest

Bacterial strains were pre-cultivated by streaking glycerol stocks onto TSA plates (0.5 × TSB, 1.2% Agar), and incubating at 28°C for 24 h. Single colonies were picked from plates and inoculated into TSB medium (0.5 × TSB), and incubated for 24 h at 28°C with 200 rpm shaking. Next, cells were harvested by centrifuging 800 μL of culture at 5,000 × g for 2 min at RT. These cells were then rinsed 3 × in sterile 10 mM MgCl_2_, and resuspended at a final OD_600_ of 1.0 in sterile 10 mM MgCl_2_.

### Phenotype Microarrays

For phenotype microarrays using PM3B (Biolog), 12 mL of inoculant was prepared comprising 10 mL of 1.2 × IF-0 (Biolog), 1.2 mL of 500 mM glucose, 600 μL of bacterial suspension (as prepared above), 120 μL of Redox Dye D (Biolog) and 80 μL of sterile water. Next, 100 μL of this inoculant (starting OD_600_ of 0.05) was loaded into each well of the phenotype microarray, which was transferred to a plate reader (Tecan Infinite Pro 100) and incubated at 28°C for 72 h with shaking (30 s continuous orbital shaking followed by 9:30 min stationary, shaking amplitude 3 mm). Tetrazolium reduction at A_590_ was measured once per 10 min cycle, without correcting for path length. Three biological replicates were conducted. Derived curves were fitted to a logistic equation using the Growthcurver program (Sprouffske and Wagner, 2016). For each well in every assay, background was subtracted by subtracting the value of the negative control (well A1) from each time point. In our hands, guanosine (well F7) gave a very high background reading and was excluded from the analysis. Wells were considered growth-positive if the carrying capacity (k) of the logistic fit was greater than A_590_ of 0.1 in at least two of the three independent biological replicates. Next, area under the curve (AUC) values for all growth-positive wells were z-score normalized within each strain, and the average value of the three replicate assays was calculated. These averaged z-score values were divided into quartiles, so the presented data represents five possible growth intensities, ranging from 0 (no growth) to 4 (highest AUC quartile).

### Metabolic Models and Computational Simulations

The EnsembleFBA workflow from Biggs and Papin (Biggs and Papin, 2017) was adapted to analyze the three studied bacterial strains. Scripts were implemented either in Matlab (Mathworks) as the original code, or adapted for Python (Python Software Foundation). Briefly, genomes were downloaded from NCBI (Agarwala et al., 2018) and uploaded to KBase (Arkin et al., 2018) where genome re-annotation and draft metabolic model reconstruction was performed. Outputted draft networks were downloaded and used as inputs for the EnsembleFBA workflow. Also inputted to Ensemble FBA were the composition of the Biolog media, and the experimentally derived growth matrices obtained from PM3B phenotype microarray. Next, 50 metabolic networks were generated for each strain, with each network being trained on 26 nitrogen substrates that supported growth and 11 nitrogen substrates that didn’t support growth, in order to perform positive and negative gapfilling. Compounds present on the phenotype microarray but not found in the ModelSEED database (Henry et al., 2010) were excluded, and a second set of simulations excluding the five N-sources used for proteomics experiments were also obtained for unbiased integration with the proteomics datasets. To evaluate the performance of EnsembleFBA for predicting growth on the different N-sources, its accuracy, precision and recall were compared to randomly generated predictions, after masking the conditions used to gapfill the individual networks to avoid bias. Metabolic activity on a given nitrogen source was estimated as the average growth rate obtained with EnsembleFBA, and weighted according to the fraction of networks in the ensemble that predicted growth. Metabolic fluxes through specific reactions were estimated by averaging the substrate flux for each reaction across all the networks in the ensemble, and weighted according to the fraction of networks where the reaction was occuring. To visualize up- or down-regulated metabolic fluxes in metabolic pathway maps, metabolic fluxes obtained by simulating growth on glutamate, serine or lysine were compared vs. ammonium, and filtered for reactions with log2 fold change >1.

### Cultivation on Individual N-Sources for Growth Assays and Proteomic Analysis

For growth assays on individual N-sources, media were based on M9 formulation (CSHL, 2010) with nutrient concentrations of: 50 mM glucose, 24 mM Na_2_HPO_4_, 11 mM KH_2_PO_4_, 4 mM NaCl, 350 μM MgSO_4_, 100 μM CaCl_2_, 50 μM Fe-EDTA, 50 μM H_3_BO_3_, 10 μM MnCl_2_, 1.75 μM ZnCl_2_, 1 μM KI, 800 nM Na_2_MoO_4_, 500 nM CuCl_2_, 100 nM CoCl_2_. To this, one nitrogen source was added at 5 mM elemental-N (i.e.,: 5 mM of ammonium, glutamate and serine, or 2.5 mM of urea and lysine). For growth assays, 20 μL of bacterial suspension (as prepared above) was inoculated into 380 μL of growth medium (starting OD_600_ of 0.05), in individual wells of a sterile 48-well plate (Corning). These plates were then transferred to a plate reader (Tecan Infinite Pro 100) and incubated at 28°C for 48 h with shaking (3 min continuous orbital shaking followed by 7 min stationary, shaking amplitude 3 mm). Culture density at OD_600_ was measured once per 10 min cycle, without correcting for path length. Four biological replicates were conducted. To obtain quantitative growth metrics, a logistic equation was fitted to measured growth curves using the Growthcurver program (Sprouffske and Wagner, 2016). To collect samples for proteomics, cultivation was identical, except that bacterial cells were harvested during the exponential growth phase. This was achieved by investigating the 48 h growth curves collected previously, and conducting each bacterial harvest at a timepoint whereby culture turbidity was between 1/4 and 3/4 of the maximum OD600 value. Harvest involved pooling of four replicate wells (total of 1.6 mL culture), followed by centrifugation at 10,000 × g for 3 min at 4°C. Supernatant was discarded, and cell pellets were rinsed twice with 900 μL of 4°C PBS via centrifugation at 10,000 × g for 3 min at 4°C. Rinsed cell pellets were then flash-frozen and stored at −80°C.

### Proteomic Sample Preparation

Cellular protein was extracted using protocols modified from Tanca et al. (2014) as well as Wessel and Flugge (1984). To frozen cell pellets, 250 μL of lysis buffer (5% SDS, 100 mM DTT, 100 mM Tris pH 7.5) was added, along with ∼100 μL of acid-washed glass beads (1 mm diameter). Samples were then incubated for 10 min on an orbital mixer at 95°C with 1,500 rpm shaking, then at −80°C for 10 min, then bead-beaten (Bead Ruptor 24, Omni International) at 5 ms^–1^ for 10 min. Next, samples were again incubated at −80°C for 10 min, then incubated for 10 min on an orbital mixer at 95°C with 1,500 rpm shaking, then bead-beaten at 5 ms^–1^ for 10 min. Finally, samples were centrifuged at 20,000 × g for 10 min at RT, and 200 μL of supernatant was transferred to a new tube. Protein was precipitated via the addition of 800 μL MeOH, 500 μL H_2_O, and 200 μL chloroform followed by centrifugation at 10,000 × g for 5 min at 4°C. The upper aqueous phase was removed and discarded, 700 μL MeOH was added to the lower organic phase and samples were centrifuged at 200,000 × g for 10 min at 4°C. Protein pellets were then rinsed twice with −20°C acetone via centrifugation at 200,000 × g for 10 min at 4°C, before being air-dried at RT for 15 min. Dried protein pellets were stored at −80°C. To solubilize protein pellets, 40 μL of solubilization buffer (8 M urea, 50 mM TEAB, 5 mM DTT) was added, and samples were incubated on an orbital mixer at 28°C for 1 h with 350 rpm mixing. Next, CAA was added to a final concentration of 30 mM, and samples were incubated on an orbital mixer at 28°C for 30 min with 350 rpm mixing in darkness. To quantify protein concentration, an aliquot of the protein extract was taken and diluted 1:8 in water, then a Bradford assay was performed on the diluted protein samples using BSA as standard. Next, 40 μg of protein extract was transferred to a new tube and incubated with 0.8 μg Lys-C for 2 h at 37°C with 350 rpm shaking. Samples were then diluted 1:8 in TEAB, 0.8 μg of trypsin was added, and samples were incubated overnight at 37°C. Next day, samples were acidified by adding formic acid to a final concentration of 1%. Peptides were then cleaned up via SPE using SDB-RP stage tips. Following elution from stage tips, peptides were dried down in a vacuum centrifuge and stored at −80°C.

### Mass Spectrometry

Digested peptides were analyzed on a QExactive Plus mass spectrometer (Thermo Scientific) coupled to an EASY nLC 1000 UPLC (Thermo Scientific). Dried peptides were resolubilized in solvent A (0.1% formic acid), and loaded onto an in-house packed C18 column [50 cm × 75 μm I.D., filled with 2.7 μm Poroshell 120 (Agilent)]. Following loading, samples were eluted from the C18 column with solvent B (0.1% formic acid in 80% acetonitrile) using a 2.5 h gradient, comprising: linear increase from 4 to 27% B over 120 min, 27–50% B over 19 min, followed by column washing and equilibration. Flow rate was at 250 nL/min. Data-dependent acquisition was used to acquire MS/MS data, whereby the 10 most abundant ions (charges 2–5) in the survey spectrum were subjected to HCD fragmentation. MS scans were acquired from 300 to 1,750 m/z at a resolution of 70,000, while MS/MS scans were acquired at a resolution of 17,500. Following fragmentation, precursor ions were dynamically excluded for 25 s.

### Label-Free Protein Quantification

Label-free quantification of protein abundance was conducted with MaxQuant v1.5.3.8 (Tyanova et al., 2016). Acquired MS/MS spectra were searched against FASTA protein sequences for the three studied bacterial strains, obtained from IMG (Chen et al., 2017). Sequences of common contaminant proteins were also included in the search database. Protein FDR and PSM FDR were set to 0.01%. Minimum peptide length was seven amino acids, cysteine carbamidomethylation was set as a fixed modification, while methionine oxidation and protein N-terminal acetylation were set as variable modifications.

### Statistical Analysis of Proteomic Data

To determine proteins that exhibited significantly different abundance between N-treatments, a statistical threshold was imposed where the MaxQuant LFQ values must differ by log_2_FC >1 and BH-*p*-value <0.05. To determine the abundance of Kegg orthologs (KOs) across bacterial strains and N-treatments, KOs annotated to proteins via IMG were matched across bacterial strains. Data were filtered to contain only the 495 KOs that were observed in at least three replicates across all five treatments in all three strains. In instances where a single strain had multiple proteins matching the same KO, the protein with the highest average MaxQuant LFQ value across all samples was taken as the representative KO for that strain. To determine the KEGG pathways that were significantly modulated at the protein abundance level between nitrogen treatments, KOs annotated onto proteins via IMG were mapped against KEGG pathways using KEGG-REST, and Fisher’s exact test was used to generate a single *p*-value for each KEGG pathway by combining the individual BH-*p*-values for all constituent proteins mapped to that pathway. Pathways were only analyzed when at least three representative proteins were detected for a single strain across all five nitrogen treatments, and pathways associated with non-bacterial processes were discarded.

## Results

We studied nitrogen metabolism in three taxonomically diverse bacterial strains isolated from roots of field-grown Arabidopsis: *Pseudomonas* sp. *Root9*, *Streptomyces* sp. *Root66D1*, and *Rhizobium* sp. *Root491*. All three strains were previously isolated in Bai et al. (2015). The strains were selected for study based on two criteria: first that they were previously shown to correspond to highly abundant taxa in the root microbiome of field-grown Arabidopsis plants (Table 1); and second that they could be successfully cultivated on a set of minimal media using different N-sources, based on a preliminary experiment with a panel of 17 rhizosphere bacterial strains (Supplementary Table S1).

**TABLE 1:** Literature information about the abundance of these three strains in the rhizosphere of field-grown Arabidopsis plants.

Strain	Relative abundance of corresponding taxonomic category in published bacterial microbiota surveys from field-grown Arabidopsis roots (Levy et al., 2018b)			Abundance rank of corresponding OTU in published bacterial microbiota surveys from field-grown Arabidopsis roots (Bai et al., 2015)			Number of phylogenetically similar strains (Levy et al., 2018b)	
			
	Taxonomic category	Lundberg et al., 2012	Bulgarelli et al., 2012	Bulgarelli et al., 2012	Schlaeppi et al., 2014	Bai et al., 2015	Number of strains in same branch of phylogenetic tree	Number of those strains that are plant-associated
*Pseudomonas* sp. *Root9*	Pseudomonas (Genus)	2%	2%	9	11	17	19	8
*Streptomyces* sp. *Root66D1*	Actinobacteria 1 (Phylum)	16%	13%	1	3	2	12	3
*Rhizobium* sp. *Root491*	Alphaproteobacteria (Class)	5%	6%	8	19	34	16	13

*(Bulgarelli et al., 2012; Lundberg et al., 2012; Schlaeppi et al., 2014; Bai et al., 2015; Levy et al., 2018b).*

Although the three strains studied here were newly isolated in Bai et al. (2015) they exhibit genetic similarities to strains with an extensive history of scientific study. To find well-characterized relatives for each of these three strains, we searched for literature information amongst similar strains that were grouped into a neighboring taxonomic position via the phylogenetic tree presented in Levy et al. (2018b). This showed that *Pseudomonas* sp. *Root9* was grouped into the same phylogenetic branch as both *Pseudomonas* sp. *WCS374* and *Pseudomonas simiae WCS417*, which were originally isolated from the rhizosphere of crop plants and repeatedly shown to exert plant-growth-promoting effects (Berendsen et al., 2015). *Streptomyces* sp. *Root66D1* is located in the same phylogenetic branch as *Streptomyces clavuligerus ATCC 27064*, originally isolated from soil and well documented to produce a wide repertoire of bioactive secondary metabolites (Medema et al., 2010). *Rhizobium* sp. *Root491* is located in the same phylogenetic branch as *Agrobacterium* sp. *H13-3*, originally isolated from Lupin rhizosphere and extensively studied as a model system for investigating chemotaxis and motility (Wibberg et al., 2011).

### Measurement and Modeling of Growth Phenotypes on Different Nitrogen Sources

**FIGURE 1:**
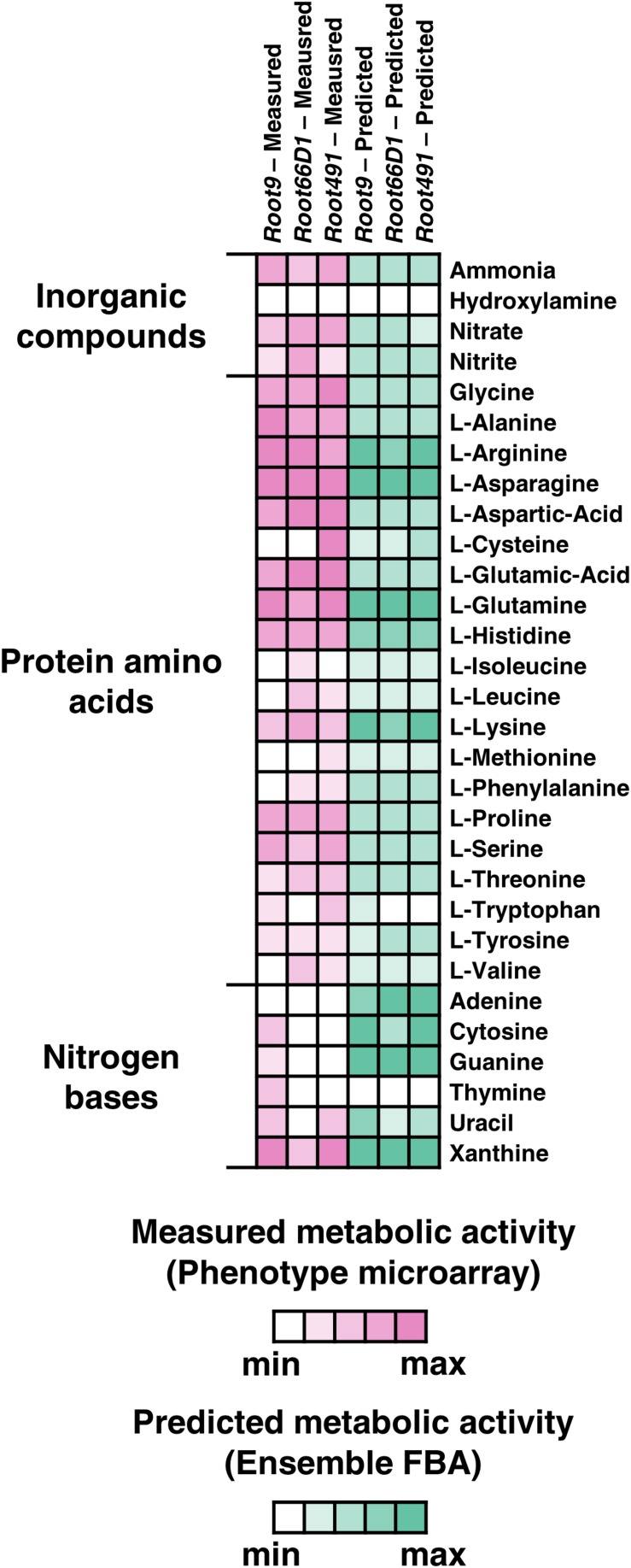
Nitrogen substrate preferences of three rhizosphere bacterial strains assessed via Phenotype Microarray and EnsembleFBA. Displayed here are results for 30 nitrogen substrates selected from the 94 tested. White boxes indicate no metabolic activity, whereas boxes with darker shades correspond to higher metabolic activity, either measured via Phenotype Microarray (pink) or predicted via EnsembleFBA (green). Metabolic activity values were z-score normalized within each strain.

First, we investigated each strain’s ability to utilize 94 diverse nitrogen sources using a phenotype microarray (BIOLOG PM3B) (Supplementary Figure S1 and Supplementary Table S2). The data reveal that all three strains can catabolize a relatively high number of substrates, with the three strains exhibiting positive growth phenotypes on 55–61 of the 94 substrates tested. This indicates that all three strains have generalistic nitrogen substrate preferences, which has been previously suggested to be a selective advantage in the rhizosphere (Lopez-Guerrero et al., 2013). In parallel, we used EnsembleFBA (Biggs and Papin, 2017) to test how accurately nitrogen substrate utilization can be computationally predicted across the three strains (Supplementary Figure S1 and Supplementary Table S2). When nitrogen substrate utilization is assessed in binary terms (growth vs. no growth), there is a good concordance between the experimental results and the computational predictions, with Ensemble FBA showing an accuracy in predicting growth in about 80% of cases for the three strains (Supplementary Table S3). However, there is a relatively poor correlation between the proxy values of metabolic activity predicted by the models vs. the experimental measurements, with a comparison of percentile rank between the datasets yielding *r*^2^ values between 0.23 and 0.5 across the three strains (Supplementary Figure S2). The accuracy of the model prediction seems to vary across different molecular classes, with good concordance for amino acids but poor concordance for nitrogen bases (Figure 1).

### Growth Curves in Batch Culture

**FIGURE 2:**
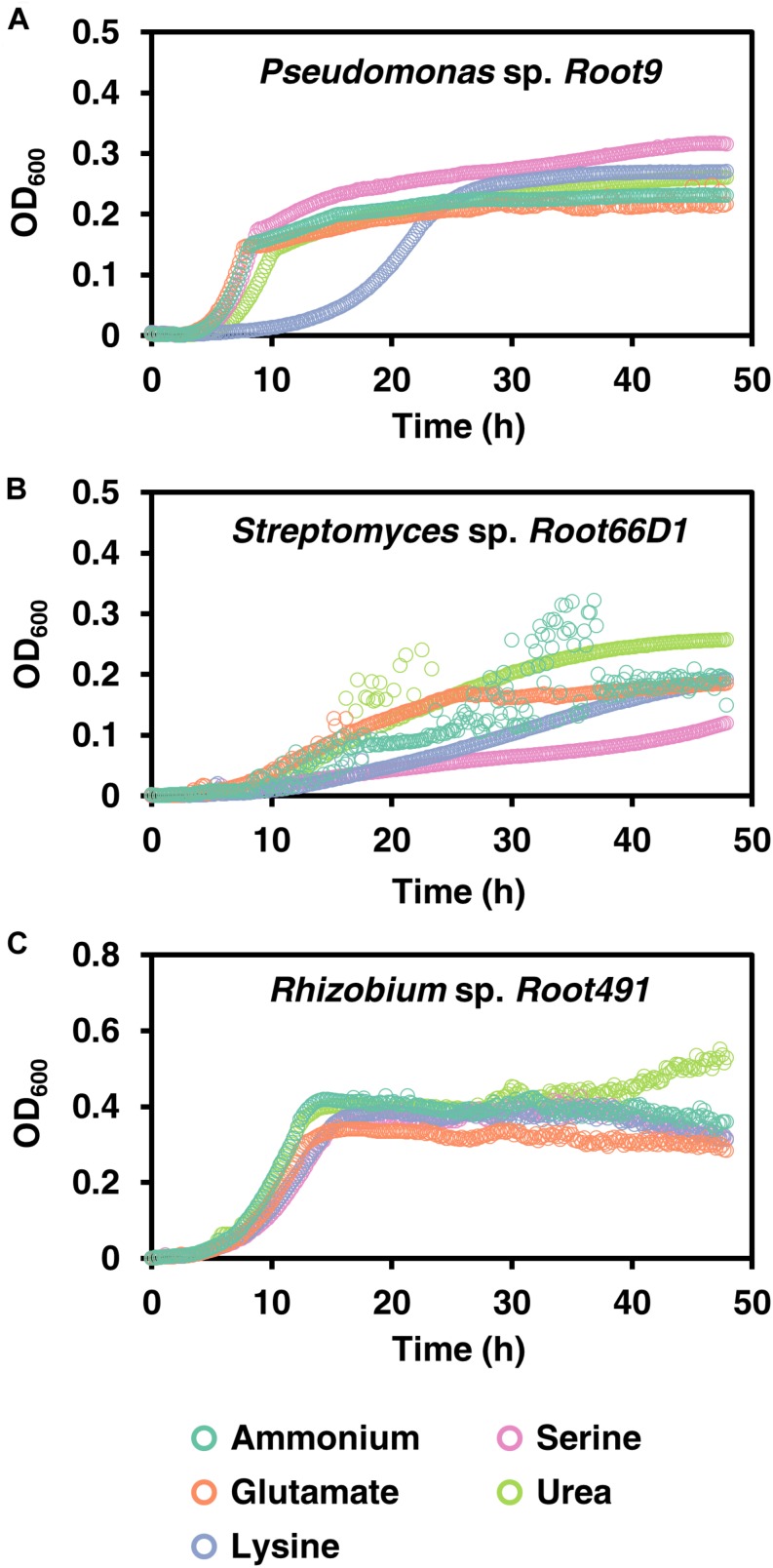
Growth curves on five nitrogen sources for three rhizosphere bacterial strains. **(A)** *Pseudomonas* sp. *Root9*, **(B)** *Streptomyces* sp. *Root66D1*, **(C)** *Rhizobium* sp. *Root491*. Cultures were grown in 48-well plates on minimal medium containing a single nitrogen source. OD_600_ (uncorrected for path length) was logged every 10 min using a plate reader.

We conducted growth curves in batch culture to further investigate the growth phenotypes of these three strains when cultivated on five selected nitrogen sources (ammonium, glutamate, lysine, serine, and urea; Figure 2 and Supplementary Table S4). The rationale for selecting these nitrogen sources is because ammonium serves as the inorganic reference, the three chosen amino acids are abundant in soils (Warren, 2014) and exhibit diverse charges (glutamate negative, lysine positive, serine neutral), while urea is a widely applied agricultural fertilizer. Nitrogen concentration in the media corresponded to 5 mM of elemental-N, which was empirically determined to be a yield-limiting nitrogen concentration for all three strains (Supplementary Figure S3), following the recommendations of Egli (2015). In *Pseudomonas* sp. *Root9*, we see that lysine nutrition elicits a long extension of the lag phase (Figure 2A). In contrast, *Rhizobium* sp. *Root491* exhibited broadly similar growth curves across all five nitrogen sources, indicative of growth homeostasis across different nutrient sources. In *Streptomyces* sp. *Root66D1*, growth rates were slower on lysine and serine compared to the other three tested nitrogen sources.

Culture turbidity measurements do not represent the absolute number of bacterial cells, particularly when comparing between different strains that may have differences in cell size or opacity. Therefore, we investigated the relationship between bacterial count (CFU/mL) vs. turbidity (OD_600_) in these three strains (Supplementary Figure S4). This showed that bacterial count is linearly correlated to OD_600_ at the turbidity values studied in Figure 2, and also that cultures of *Rhizobium* sp. *Root491* contain many more bacterial cells compared to the other two strains when plated at the same OD_600_ value.

### Proteome Remodeling in Response to Different Nitrogen Sources

**FIGURE 3:**
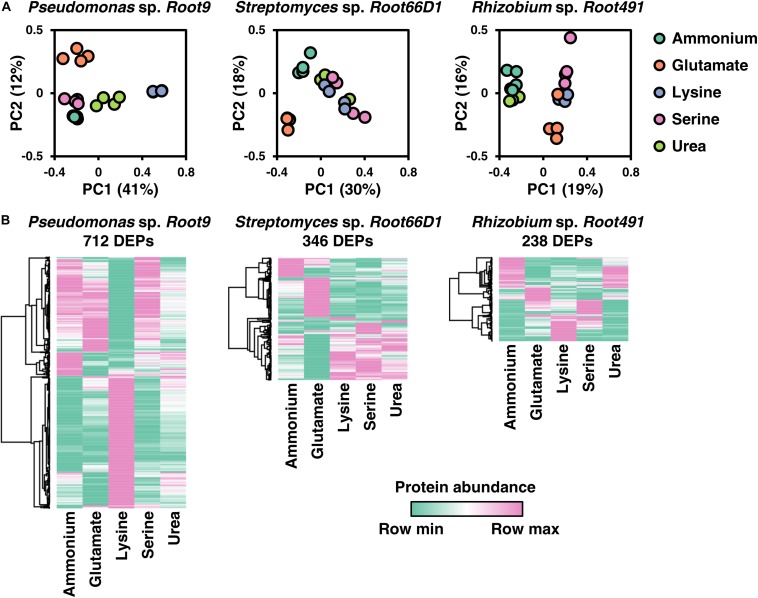
Overview of proteome composition in three rhizosphere bacterial strains when cultivated on five nitrogen sources. **(A)** Principal component analysis (PCA) of the five different nitrogen sources for each strain. **(B)** Heat maps of protein abundance for differentially expressed proteins (DEPs) for each of the three strains. To define DEPs, protein abundance in one condition was compared to its abundance in the other four conditions. If in any of these 10 comparisons, a protein has a log2FC >1 and a BH-*p*-value <0.05, then it is considered a DEP. Only DEPs that were detected in at least three replicates for all five nitrogen treatments are included in the heatmaps. Rows were clustered using Pearson’s correlation coefficient.

The main aim of this study was to define systems-level differences in cellular proteome composition in three rhizosphere bacterial strains cultivated on five different nitrogen sources. Therefore, bacteria were cultivated on the same nitrogen sources shown in Figure 2 (ammonium, glutamate, lysine, serine, and urea), cells were harvested during the exponential growth phase, and cellular protein composition analyzed using label-free quantitative proteomics. A numerical summary of protein IDs is shown in Table 2, a visual overview of the derived results is shown in Figure 3, volcano plots for all 10 pairwise comparisons across all three strains are shown in Supplementary Figures S5–S7, and the MaxQuant abundance values for all detected proteins are given in Supplementary Table S5.

**TABLE 2:** Summary of label free quantitative proteomic data for three rhizosphere bacterial strains cultivated on five different nitrogen sources.

	*Pseudomonas* sp. *Root9*	*Streptomyces* sp. *Root66D1*	*Rhizobium* sp. *Root491*
Proteins encoded in genome	5,871	6,744	5,225
Proteins observed in any treatment (*n* ≥ 3)	3,117	2,552	3,358
Proteins observed in all five treatments (*n* ≥ 3), abundance significant between any 2 (log2FC > 1, BH *p*-value < 0.05)	712	346	238
Proteins observed in ≥ 1 treatment (*n* ≥ 3), but undetected in ≥ 1 other treatment (*n* = 0)	548	168	397

The most noticeable observation in these quantitative proteomic datasets is that the three different bacterial strains exhibit widely divergent protein-level responses to the same nitrogen source. This is best illustrated by the differential responses to lysine nutrition, which elicited widespread alterations to the proteome of *Pseudomonas* sp. *Root9* but relatively fewer protein-level changes in the other two studied strains (Figure 3). By matching the specific enzymes that were up-regulated by lysine nutrition onto metabolic pathways for lysine degradation, the proteomic data indicate that lysine degradation in *Pseudomonas* sp. *Root9* proceeds via the δ-aminovalerate pathway, whereas *Rhizobium* sp. *Root491* utilizes the saccharopine pathway. Two other nitrogen sources that elicit divergent proteomic responses between the strains are serine and urea. Appraising how the different strains respond to serine nutrition, the proteomic datasets show that *Pseudomonas* sp. *Root9* induced a very small set of proteins including serine dehydratase, which yields ammonium in one enzymatic step as well as pyruvate that can be quickly assimilated in the TCA cycle. In contrast, the other two studied strains exhibited widespread proteome changes between ammonium vs. serine nutrition, but no upregulation of serine dehydratase, potentially indicating that the assimilated serine must be distributed through multiple elements of the metabolic network requiring a wider modulation of protein expression. Regarding urea, both *Streptomyces* sp. *Root66D1* and *Rhizobium* sp. *Root491* showed zero proteins that were differentially expressed between ammonium vs. urea treatment, whereas this comparison in *Pseudomonas* sp. *Root9* elicited 126 differentially expressed proteins.

### Orthologous Proteins and Metabolic Pathways Modulated by Nitrogen Nutrition

**FIGURE 4:**
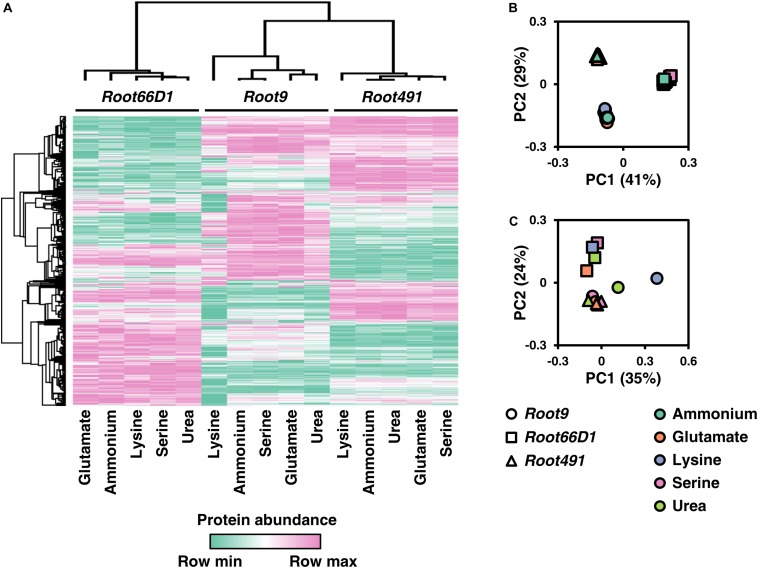
Comparison of protein abundance values for 495 KOs (Kegg orthologs) across three rhizosphere bacterial strains cultivated on five nitrogen sources. **(A)** Heat map of KO abundance across the three rhizosphere bacterial strains cultivated under five nitrogen sources. **(B)** Principal component analysis (PCA) of KO abundance across the three rhizosphere bacterial strains cultivated under five nitrogen sources. **(C)** Principal component analysis (PCA) of KO abundance across the three rhizosphere bacterial strains for the four organic nitrogen sources, when KO abundance was normalized to ammonium (inorganic reference). The KOs annotated to proteins via IMG were matched across the proteomic dataset for the three bacterial strains. Data was filtered to contain only the 495 KOs that were observed in all four replicates across all five treatments in all three strains. MaxQuant LFQ abundance values were z-score normalized within each strain. Rows and columns were clustered using Pearson’s correlation coefficient.

To enable inter-strain comparisons of the label-free quantitative proteomic data acquired from the three taxonomically diverse rhizosphere bacterial strains, we utilized cross-species gene annotation via KEGG orthologs (Kanehisa et al., 2016). We selected individual proteins that represent the 495 KEGG orthologs which were detected in all five treatments across all three strains, and visualize the abundance of these representative orthologs using a heatmap and PCAs in Figure 4, with numerical data provided in Supplementary Table S6. As can be seen in Figures 4A,B, the samples group together according to the three bacterial strains rather than the five nitrogen sources. This indicates that the baseline differences in strain-specific proteome composition are much greater than any treatment-induced differences elicited by nitrogen nutrition. In Figure 4C we plot a PCA of these 495 KEGG orthologs when protein abundance in the four organic nitrogen sources is normalized vs. the inorganic nitrogen source ammonium. This shows that lysine nutrition in *Pseudomonas* sp. *Root9* elicits a proteomic response that is qualitatively different compared to the strain-medium combinations profiled in this study.

**FIGURE 5:**
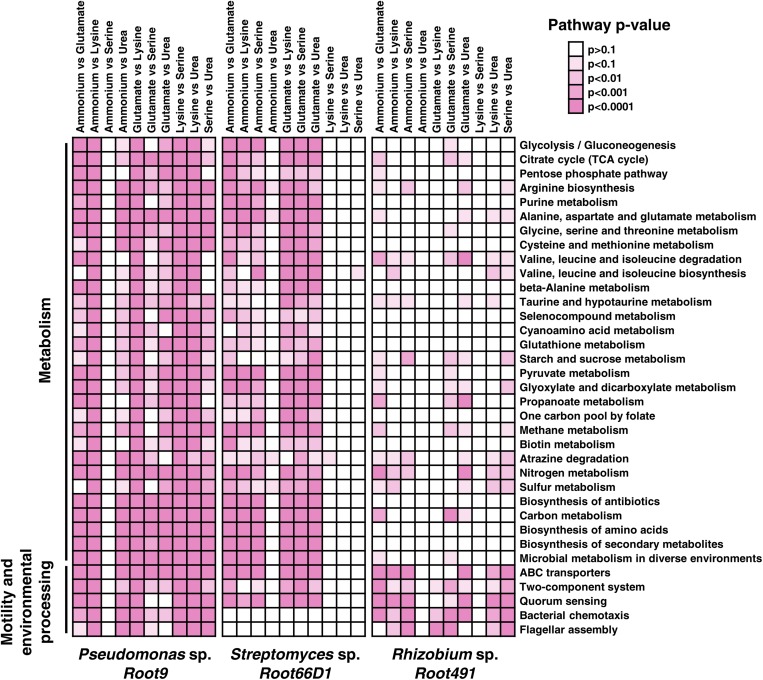
Assessment of KEGG pathways that were modulated at the protein abundance level between different nitrogen treatments. Kegg orthologs annotated to proteins via IMG were matched to KEGG pathways, and Fisher’s exact test was used to determine the statistical significance of pathway modulation between two nitrogen treatments. Darker shades of pink represent lower *p*-values via Fisher’s exact test. Pathways with fewer than three identified proteins were excluded from analysis. This figures shows the 30 pathways with the highest number of significantly differences between treatments (*p* < 0.01), data for all ∼100 pathways are in Supplementary Table S7.

Our next step was to analyze which specific KEGG pathways were modulated according to nitrogen treatment in the three strains. In Figure 5, we show the results of Fisher’s exact test to determine whether the constituent proteins of 30 KEGG pathways exhibited altered abundance profiles in the 10 pairwise comparisons between different nitrogen sources. Numerical data for all 126 tested pathways compared is provided in Supplementary Table S7. Looking at the specific pathways modulated by nitrogen nutrition across the three strains, it seems that *Rhizobium* sp. *Root491* undergoes fewer alterations to KEGG pathways related to metabolism, but instead exhibits extensive modulation to ABC transporters. This indicates that *Rhizobium* sp. *Root491* utilizes different transport mechanisms to assimilate diverse nitrogen sources into a relatively stable metabolic network. For *Pseudomonas* sp. *Root9* and *Streptomyces* sp. *Root66D1*, we see that many of the pairwise comparisons are characterized by widespread modulation to all KEGG pathways, indicating that extensive proteome remodeling has taken place between the different nitrogen sources.

Next, we compared the metabolic flux distributions outputted from EnsembleFBA vs. the differentially expressed proteins identified in the quantitative proteomic datasets (Supplementary Tables S8–S14). To visualize how nitrogen source affects protein abundance and computationally predicted fluxes, we used the Interactive Pathway Explorer to map KEGG orthologs and reactions onto the KEGG map “Metabolic Pathways” (Darzi et al., 2018). Visualizations for each of the three amino acid treatments (glutamate, lysine and serine) in pairwise comparisons vs. ammonium were produced for both the proteomic data (Supplementary Figure S8) and also the computational modeling data (Supplementary Figure S9). Overall, it is evident that a similar set of metabolic pathways have been mapped in both the experimental and computational approaches, with good coverage of glycolysis, TCA cycle, and amino acid metabolism. However, there is relatively little concordance between the differentially regulated metabolic steps identified by the proteomics data vs. the differentially regulated fluxes outputted by EnsembleFBA. For instance, EnsembleFBA predicted that *Rhizobium* sp. *Root491* will exhibit widespread differences in metabolic flux distributions between ammonium vs. amino acid nitrogen sources, but this contrasts against the proteomic measurements that recorded a relatively small set of metabolic proteins that showed significant changes in abundance between these conditions. Furthermore, the proteomic data show that lysine nutrition elicits significant modifications to lipid metabolism in *Pseudomonas* sp. *Root9*, whereas many of the reaction steps in lipid metabolism are absent from the EnsembleFBA flux distributions.

### Proteins Correlated to the PII Protein of the Nitrogen Stress Response

**FIGURE 6:**
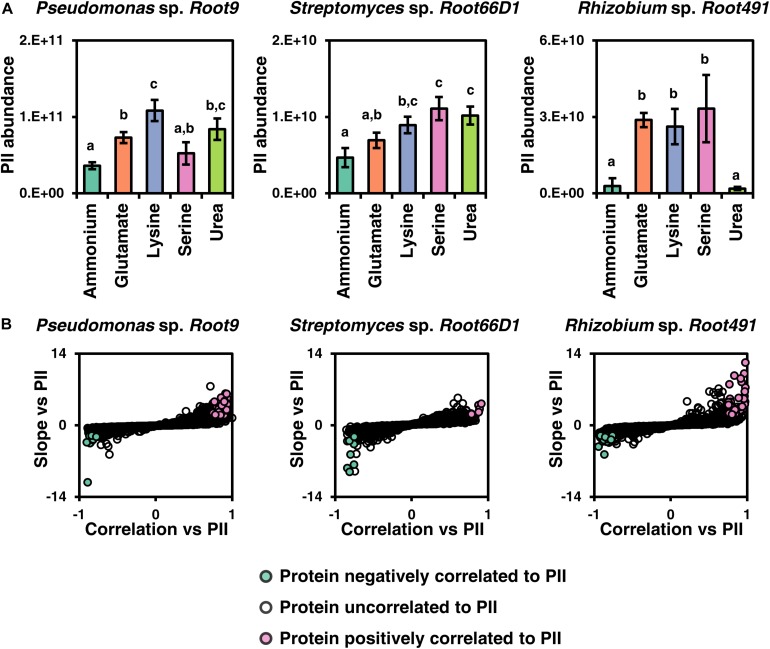
Investigating proteins correlated to the abundance of nitrogen stress response component PII. **(A)** Abundance of the PII protein across five nitrogen treatments in three rhizosphere bacterial strains. Different letters above data series indicate *p* < 0.05 following two-way ANOVA and Tukey’s HSD test. **(B)** Plots to highlight proteins that are positively or negatively correlated to PII according to their abundance values across five nitrogen treatments. Y-displays the slope of the linear fit (z-score normalized) between protein abundance vs. the abundance of PII protein, and X-axis displays correlation between protein abundance vs. PII abundance. If a protein has a correlation higher than 0.75 and a slope higher than 2, it is deemed positively correlated, whereas if a protein has a correlation lower than 0.75 and a slope lower than –2, it is deemed negatively correlated to PII.

Analyzing the quantitative proteomics data, we noticed that the different nitrogen sources often elicited changes in the abundance of proteins involved in the well-characterized nitrogen stress response, such as GlnK (PII protein), amtB (ammonium transporter), and GlnA (glutamine synthetase) (van Heeswijk et al., 2013). Therefore, we postulated that our dataset may allow us to discover new proteins that are regulatory targets of the nitrogen stress response in less studied bacterial taxa. We first analyzed the abundance of PII, a well characterized protein of the nitrogen stress response that exhibited significantly different abundance values between certain nitrogen treatments in all three strains (Figure 6A). Next, we assessed which other proteins in the dataset were correlated to PII in terms of protein abundance, by plotting their correlation against PII on the x-axis and the slope of this correlation on the y-axis (Figure 6B, numerical data in Supplementary Table S15). These analyses show that *Rhizobium* sp. *Root491* shows the highest nitrogen stress response under these nitrogen treatments, with all three amino acid treatments leading to dramatic increases in the abundance of the PII protein, and also with many more proteins positively correlated to PII abundance in Rhizobium sp. Root491 compared to the other two strains. Looking at the identity of proteins whose abundance was correlated to PII in *Rhizobium* sp. *Root491*, we see that 10 proteins controlled by the exo operon that conduct the synthesis and export of extracellular polysaccharides are positively correlated to PII abundance (Supplementary Table S15). Analogous findings have been reported via genetic manipulation of *V. vulnificus* and *S. meliloti*, with knockout of nitrogen stress response elements NtrC and NtrX resulting in reduced production of extracellular polysaccharides (Kim et al., 2009; Wang et al., 2013). In *Pseudomonas* sp. *Root9*, the data point that exhibits a strong negative correlation to PII is an NADP-dependent glutamate dehydrogenase (Supplementary Table S15), previously shown to be a target of NtrC-driven transcriptional repression in *P. putida* (Hervas et al., 2010).

## Discussion

Differential nitrogen treatments are a classical experimental manipulation in microbiology, but the majority of molecular knowledge about bacterial nitrogen metabolism has been acquired in *E. coli* (van Heeswijk et al., 2013). To deepen our knowledge of nitrogen metabolism in the rhizosphere microbiome, this study analyzes nitrogen substrate utilization in three taxonomically diverse bacterial strains previously isolated from field-grown Arabidopsis roots (Bai et al., 2015). The three strains represent taxa that are consistently detected as core members of the plant microbiome: Pseudomonas, Streptomyces and Rhizobium (Levy et al., 2018a). Using label-free quantitative proteomics, we document hundreds of proteins in each strain that exhibit differential abundance values between nitrogen sources. These protein-level measurements provide new information to the field of bacterial physiology, because other high-throughput studies of bacterial nitrogen substrate utilization have typically used transcriptomic techniques (Yurgel et al., 2013). Furthermore, we integrate experimental data with computational models, using the EnsembleFBA method to test how accurately metabolic phenotypes can be computationally predicted from a minimal set of experimental data. Our results show that the three strains exhibit diverse metabolic responses to different nitrogen nutrition regimes, with a summary of key results presented in Supplementary Table S16).

There is a longstanding appreciation that amino acids play a significant role in the nutrition of rhizosphere bacterial strains (Lochhead and Thexton, 1947). Amino acids are an important component of the soil nitrogen cycle, derived from diverse sources such as depolymerization of soil bound protein and also from plant rhizodeposition (Moe, 2013). Microbial metabolism of amino acids in the rhizosphere is related to plant productivity, because microbial mineralization of organic nitrogen can boost plant nutrition (van der Heijden et al., 2008) while the microbial uptake of amino acids is one mechanism used by plants to recruit specific strains into the rhizosphere microbiome (Zhalnina et al., 2018). The data presented here could potentially assist future efforts to manipulate the rhizosphere microbiome for altered metabolism of amino acids. For instance, our data in *Pseudomonas* sp. *Root9* implicate serine dehydratase as an important protein for degradation of serine, and measurements in *Rhizobium* sp. *Root491* position saccharopine dehydrogenase as important for degradation of lysine. Perhaps bacterial strains with high activities of these two enzymes could be recruited to the rhizosphere to promote faster rates of amino acid mineralization. In *Streptomyces* sp. *Root66D1*, amino acid nutrition results in upregulation of dozens of proteins, but very few of these are classically recognized as being involved in amino acid degradation. Compared to other bacterial taxa, there is generally less knowledge about nitrogen metabolism in Gram-positive Streptomyces (Amon et al., 2010) so the uncharacterized proteins shown to be differentially expressed under amino acid nutrition in *Streptomyces* sp. *Root66D1* could be targets for future studies investigating their biochemical function. In *Rhizobium* sp. *Root491*, we document that this strain grows quickly on three chemically diverse amino acids, and also that dozens of ABC transporter proteins exhibit altered abundance values under amino acid nutrition. Previous work in *E. coli* has shown amino acids such as glutamate and arginine serve as poor sole nitrogen sources for enteric bacteria, with this phenotype being underpinned by slow rates of amino acid transport and catabolism (Wang et al., 2016). Perhaps the protein network that undertakes amino acid transport and catabolism in *Rhizobium* sp. *Root491* could serve as a template for engineering other bacterial strains to grow rapidly on amino acids as a sole nitrogen source. Interesting candidate proteins include components of the “peptide/nickel transport system,” because the *Rhizobium* sp. *Root491* genome encodes significantly more of these genes compared to the other two strains, and the proteomics data show that several of them are dramatically upregulated by amino acid nutrition.

This study was largely descriptive, but the derived results provide a useful basis for subsequent hypothesis-driven experiments. For instance, the uncharacterized proteins upregulated under distinct N-sources in all three strains are good candidates for reverse genetic studies to elucidate their roles in nitrogen metabolism. Also, future experiments could investigate why lysine nutrition elicits a longer lag phase and dramatic proteome remodeling in *Pseudomonas* sp. *Root9*, potentially with the hypothesis that lysine exerts a signaling function in this strain. Furthermore, all three strains show extensive modulation to their membrane transport networks under certain nitrogen regimes, which suggests that nitrogen source could affect metabolite excretion profiles.

There is increasing interest in combining experimental and computational approaches to analyze microbial metabolism, with the long-term goal of quantitatively predicting the behavior of microbial communities (Succurro et al., 2017). Metabolic modeling is rapidly progressing as a powerful computational tool to explore the metabolic capacities of bacteria. However, the main limitation that prevents modeling approaches from being applied to diverse bacterial strains is the need to obtain a highly curated genome-scale metabolic model for each strain of interest. This process of model curation still requires a significant amount of manual inspection and relies heavily on accurate genome annotation (Arkin et al., 2018). In the present study, we used EnsembleFBA (Biggs and Papin, 2017) to produce metabolic models for three diverse bacterial strains using a minimal set of experimental information. We compared the derived models vs. experimental data by assessing how accurately they can predict growth phenotypes and proteome remodeling across different nitrogen sources. This showed that EnsembleFBA gives relatively accurate predictions of nitrogen substrate utilization, with binary phenotypes (growth vs. no growth) correctly predicted in around 80% of cases. However, there was only an intermediate correlation between the proxy values of metabolic activity predicted by the model vs. the experimentally acquired measurements (*r*^2^: 0.23–0.50), and a relatively poor concordance between the differential fluxes predicted by the model vs. the differentially expressed proteins identified via proteomics. We present two potential interpretations for these inaccurate predictions. First, there is no straightforward relationship between enzymatic flux and protein abundance, because the catalysis rate of many enzymes is not only regulated via abundance but also by other factors including post-translational modifications, allosteric regulators or the relative concentrations of substrates and products (Gerosa and Sauer, 2011). Second, our models used the same biomass definition that Biggs and Papin used for their EnsembleFBA analyses of *Pseudomonas* and *Streptococcus* (Biggs and Papin, 2017). Although efforts have been made to define a general biomass composition for bacteria (Xavier et al., 2017) inaccuracies of this definition can decrease the predictive power of metabolic models. Therefore, one potential pathway to improve model accuracy would involve measuring the biomass composition for all genotypes and treatments under study. Despite these limitations, our work shows that EnsembleFBA shows potential for predicting nitrogen substrate utilization across diverse bacterial strains, using minimal experimental data and requiring no manual curation of the model.

## Conclusion

Methodologically, this study showcases the power of two approaches for studying microbial N-metabolism. First, we show that EnsembleFBA offers a streamlined pathway to predict bacterial nitrogen substrate preferences, and second, we show that label-free quantitative proteomics can be used to dissect the metabolic pathways deployed by bacteria to utilize different nitrogen sources. The major biological conclusion from our study is that the three bacterial strains exhibit diverging proteomic responses to a common set of five nitrogen sources. This means that N utilization is a strain-by-strain event that needs to be assessed individually, which adds further complexity to the challenge of generating a predictive understanding of the rhizosphere microbiome. Extrapolating our results to predict the metabolic fate of nitrogen in a field setting, then it is plausible to expect that some fraction of the nitrogen present in the rhizosphere would be metabolized via the pathways described here, because these strains correspond to taxa that are highly abundant in the bacterial microbiome in field-grown Arabidopsis roots. However, these bacterial strains only represent a small fraction of the total pool of species competing for rhizosphere N-nutrients, which also includes fungi, archaea, and the plant itself. Therefore, other techniques would need to be applied to enable the quantitative prediction of nitrogen fluxes in the rhizosphere. For example, useful information could be acquired by tracking the fate of isotopically labeled nitrogen delivered into the rhizosphere, coupled with metagenomics to identify how nitrogen source affects microbiome composition, and also metatransciptomics or metaproteomics to define metabolic pathways that are upregulated by responsive members of the rhizosphere microbial community.

## Data Availability Statement

All LC-MS/MS files and MaxQuant outputs have been uploaded to ProteomXchange and can be accessed via PRIDE under the accession PXD011436 (http://www.ebi.ac.uk/pride/archive/projects/PXD011436). Details of the EnsembleFBA workflow are available at: https://github.com/asuccurro/ensembleFBA, and the KBase narrative is available at https://narrative.kbase.us/narrative/ws.37070.obj.1. Interactive maps of metabolic pathways modulated between amino acid treatments can be viewed at: https://pathways.embl.de/shared/rjacoby.

## Author Contributions

RJ performed the laboratory experiments. AS performed the computational modeling. SK supervised the project. All authors have read and approved the final manuscript.

## Conflict of Interest

The authors declare that the research was conducted in the absence of any commercial or financial relationships that could be construed as a potential conflict of interest.
