# Supplementary figures and images for: Nitrogen Substrate Utilization in Three Rhizosphere Bacterial Strains Investigated Using Proteomics
Source: Front Microbiol. 2020 Apr 28;11:784. doi: 10.3389/fmicb.2020.00784 (PMC7198800; doi:10.3389/fmicb.2020.00784)

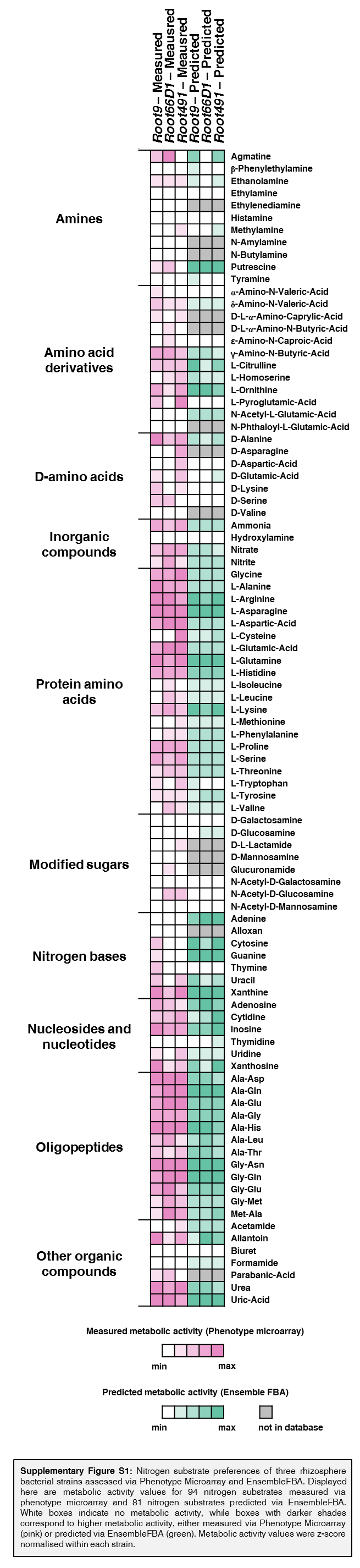

Supplement: Supplementary file 1 [file Image_1.TIF]

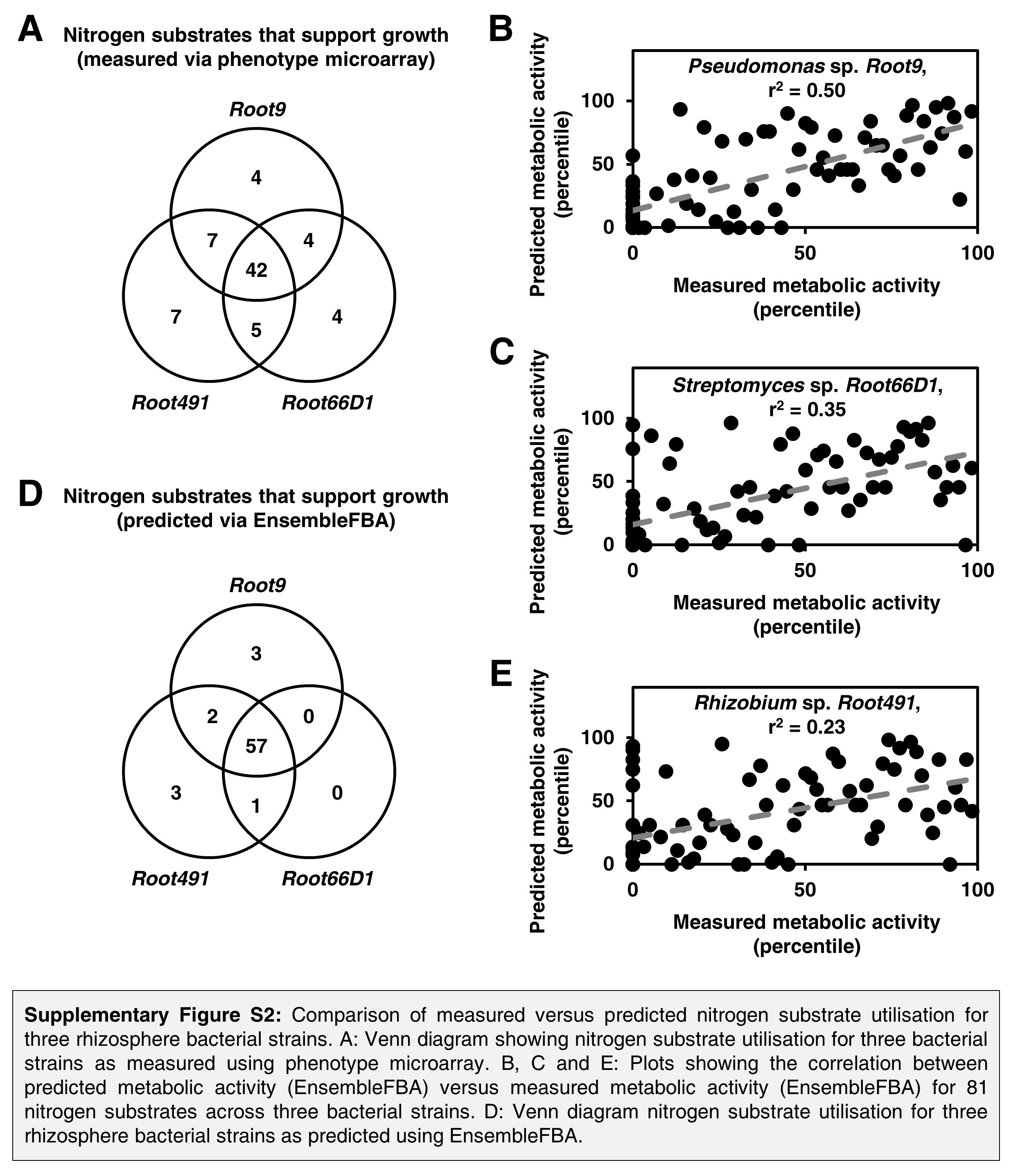

Supplement: Supplementary file 2 [file Image_2.TIF]

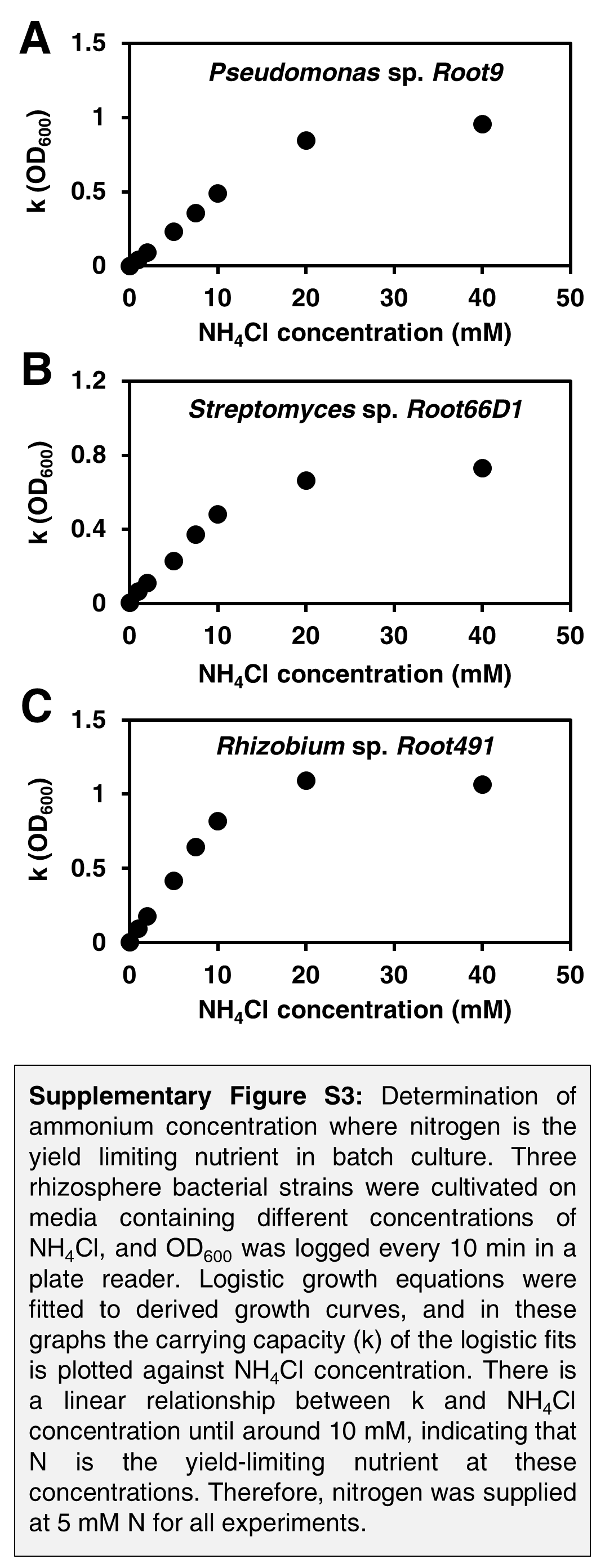

Supplement: Supplementary file 3 [file Image_3.TIF]

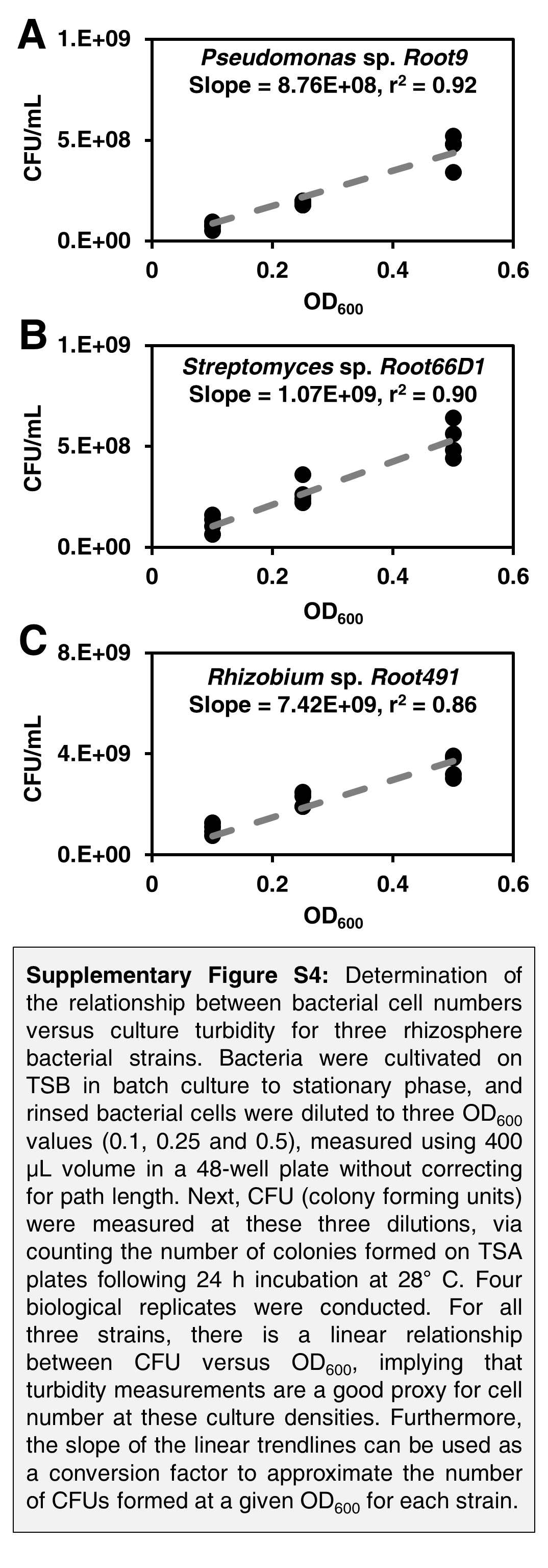

Supplement: Supplementary file 4 [file Image_4.TIF]

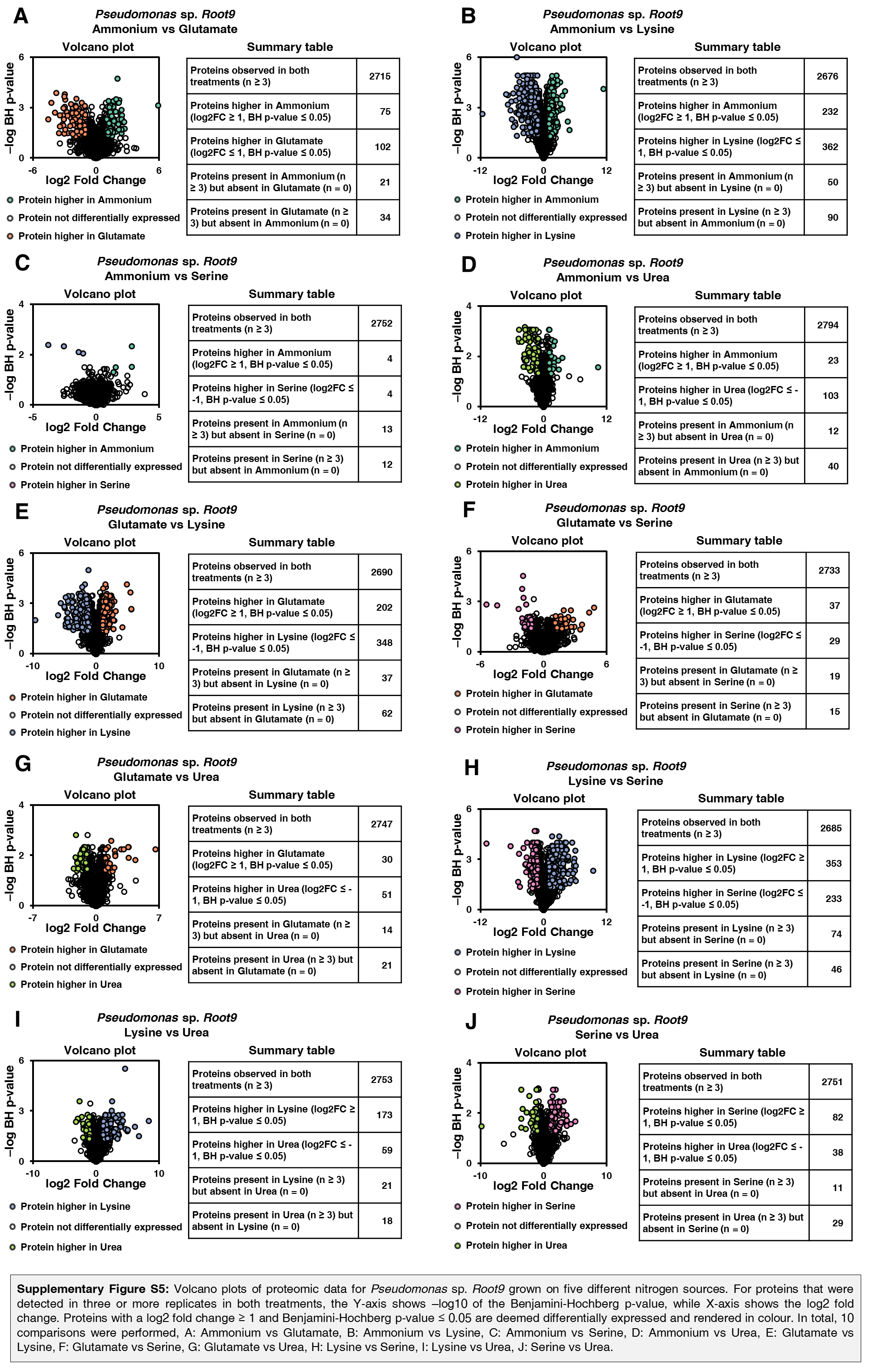

Supplement: Supplementary file 5 [file Image_5.TIF]

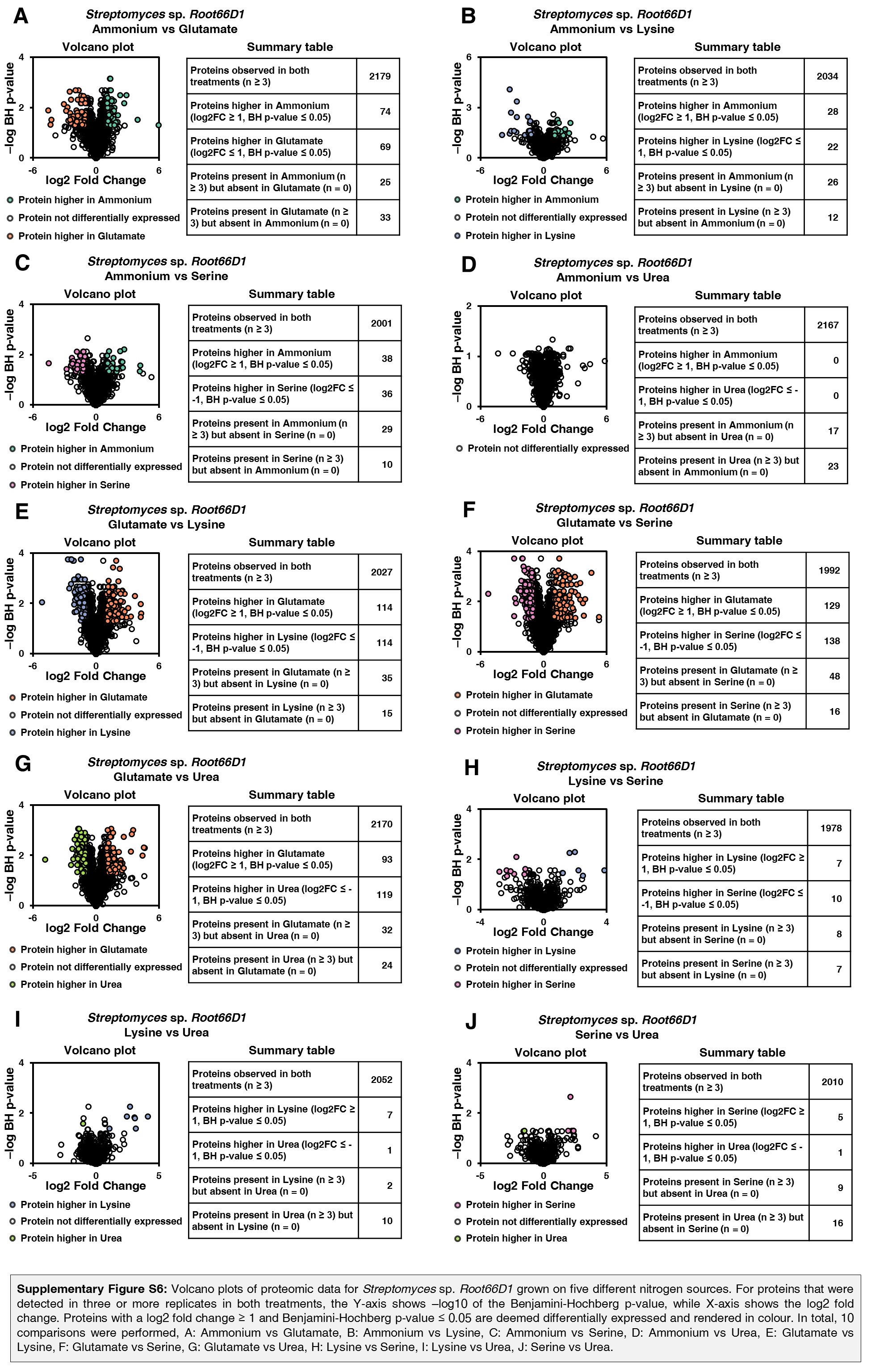

Supplement: Supplementary file 6 [file Image_6.TIF]

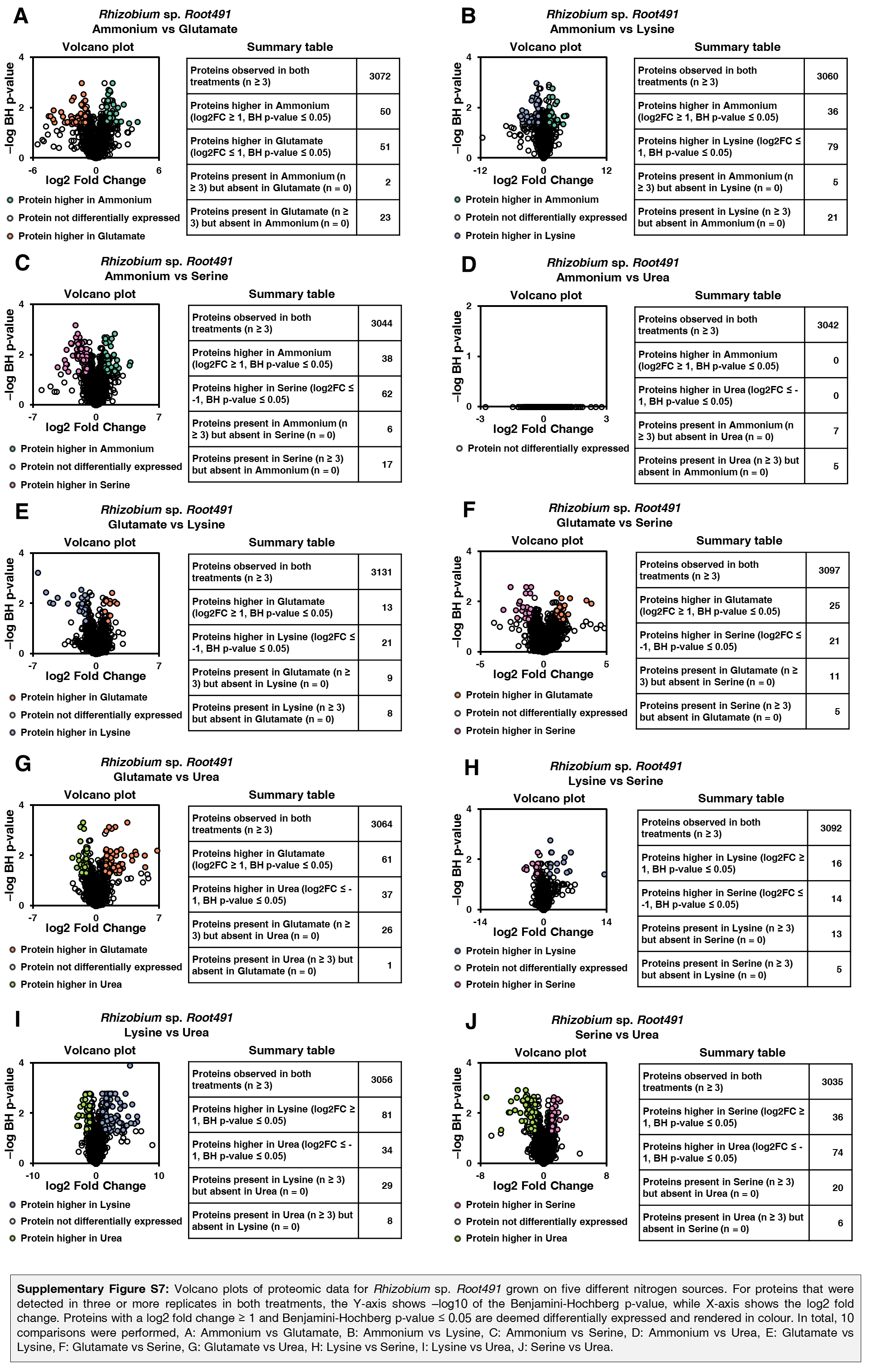

Supplement: Supplementary file 7 [file Image_7.TIF]

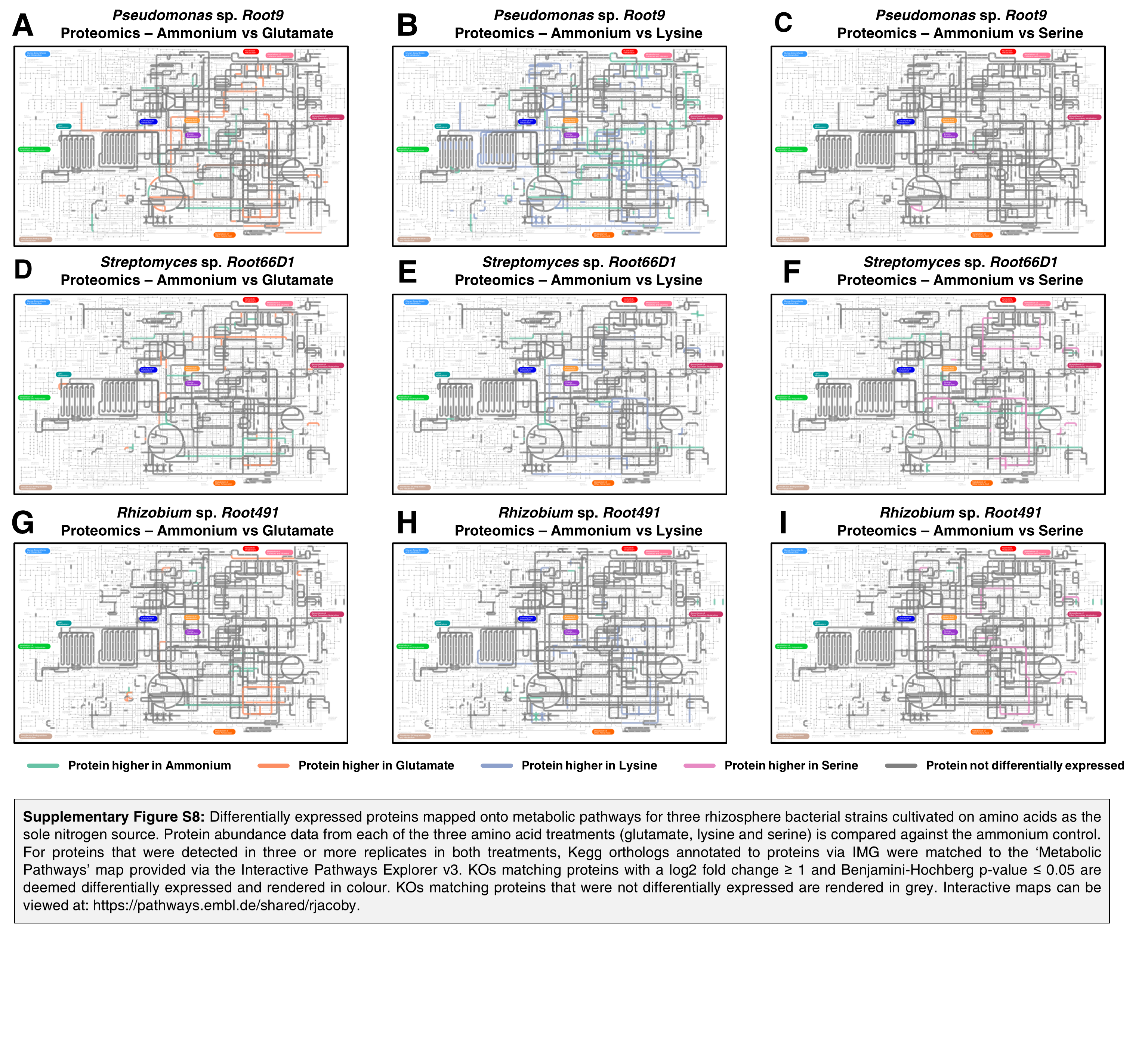

Supplement: Supplementary file 8 [file Image_8.TIF]

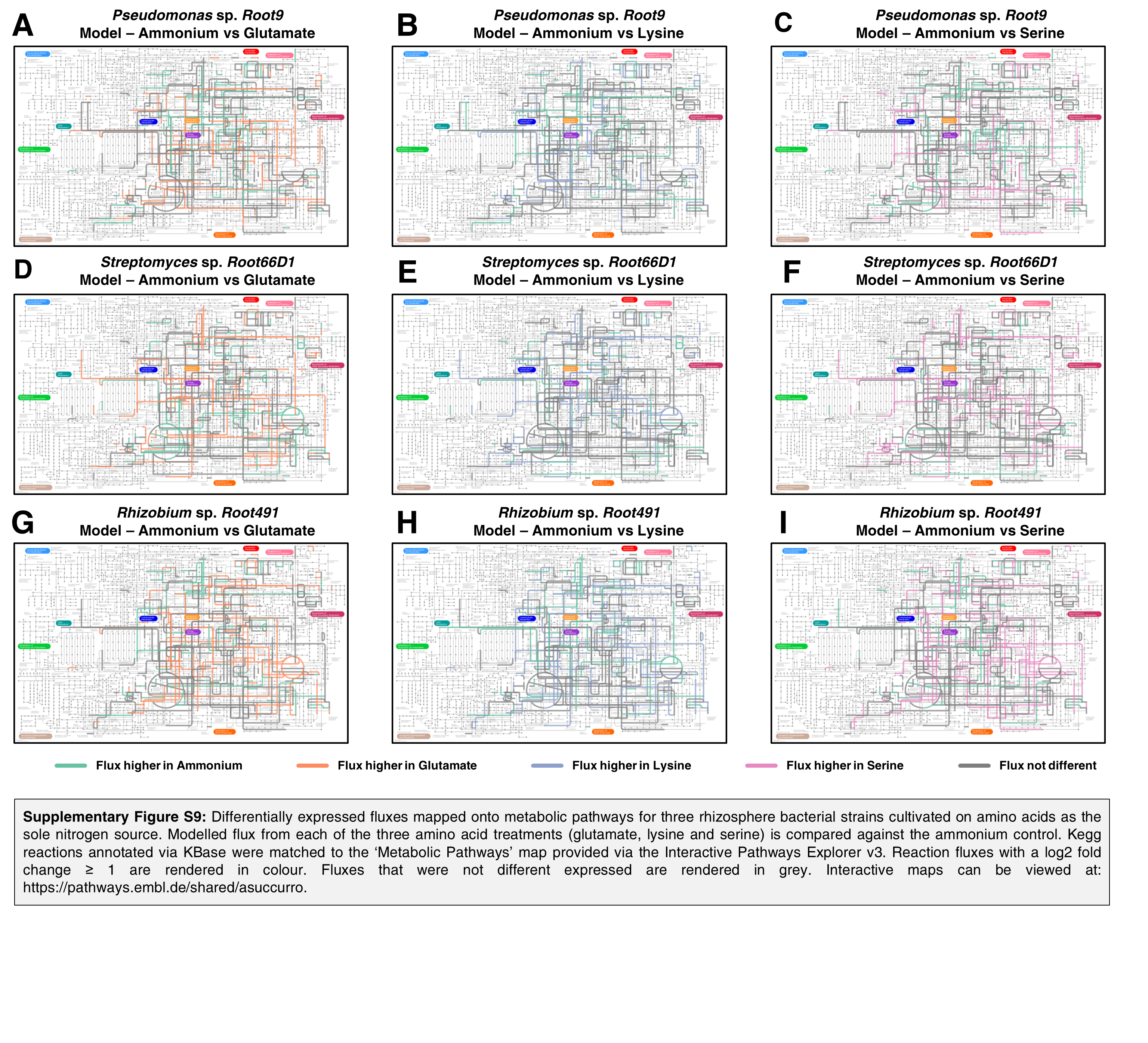

Supplement: Supplementary file 9 [file Image_9.TIF]
